# Decreasing Opioid Addiction and Diversion Using Behavioral Economics Applied Through a Digital Engagement Solution: Protocol for a Randomized Controlled Trial

**DOI:** 10.2196/52882

**Published:** 2024-03-08

**Authors:** Rubina Fatima Rizvi, Jamee Ann Schoephoerster, Sagar Satish Desphande, Michael Usher, Andy Elaine Oien, Maya Marie Peters, Matthew Scott Loth, Matthew William Bahr, Steffen Ventz, Joseph Stephen Koopmeiners, Genevieve B Melton

**Affiliations:** 1 Division of Computational Health Sciences Department of Surgery University of Minnesota Minneapolis, MN United States; 2 Center for Learning Health System Sciences University of Minnesota Minneapolis, MN United States; 3 University of Minnesota Medical School Minneapolis, MN United States; 4 Division of Plastic and Reconstructive Surgery Department of Surgery University of Minnesota Minneapolis, MN United States; 5 M Health Fairview Systems Minneapolis, MN United States; 6 Division of Biostatistics School of Public Health University of Minnesota Minneapolis, MN United States; 7 Division of Colon and Rectal Surgery Department of Surgery University of Minnesota Minneapolis, MN United States

**Keywords:** opioid abuse, opioid naïve patients, opioid addiction, behavioral economics, nudges, MyChart, personal health record, post-operative care, opioid, opioid use, randomized controlled trial, RCT, behavioral economics, digital engagement, health crisis, overdose, acute pain, pain, tool, tools, phone app, website, application

## Abstract

**Background:**

Despite strong and growing interest in ending the ongoing opioid health crisis, there has been limited success in reducing the prevalence of opioid addiction and the number of deaths associated with opioid overdoses. Further, 1 explanation for this is that existing interventions target those who are opiate-dependent but do not prevent opioid-naïve patients from becoming addicted.

**Objective:**

Leveraging behavioral economics at the patient level could help patients successfully use, discontinue, and dispose of their opioid medications in an acute pain setting. The primary goal of this project is to evaluate the effect of the 3 versions of the Opioid Management for You (OPY) tool on measures of opioid use relative to the standard of care by leveraging a pragmatic randomized controlled trial (RCT).

**Methods:**

A team of researchers from the Center for Learning Health System Sciences (CLHSS) at the University of Minnesota partnered with M Health Fairview to design, build, and test the 3 versions of the OPY tool: social influence, precommitment, and testimonial version. The tool is being built using the Epic Care Companion (Epic Inc) platform and interacts with the patient through their existing MyChart (Epic Systems Corporation) personal health record account, and Epic patient portal, accessed through a phone app or the MyChart website. We have demonstrated feasibility with pilot data of the social influence version of the OPY app by targeting our pilot to a specific cohort of patients undergoing upper-extremity procedures. This study will use a group sequential RCT design to test the impact of this important health system initiative. Patients who meet OPY inclusion criteria will be stratified into low, intermediate, and high risk of opiate use based on their type of surgery.

**Results:**

This study is being funded and supported by the CLHSS Rapid Prospective Evaluation and Digital Technology Innovation Programs, and M Health Fairview. Support and coordination provided by CLHSS include the structure of engagement, survey development, data collection, statistical analysis, and dissemination. The project was initially started in August 2022. The pilot was launched in February 2023 and is still running, with the data last counted in August 2023. The actual RCT is planned to start by early 2024.

**Conclusions:**

Through this RCT, we will test our hypothesis that patient opioid use and diverted prescription opioid availability can both be improved by information delivery applied through a behavioral economics lens via sending nudges directly to the opioid users through their personal health record.

**Trial Registration:**

ClinicalTrials.gov NCT06124079; https://clinicaltrials.gov/study/NCT06124079

**International Registered Report Identifier (IRRID):**

PRR1-10.2196/52882

## Introduction

Opioid-related overdose and death rates continue to soar in all age groups [1-3]. Today, it is the leading cause of unintentional, injury-related death in the United States [4]. As of 2020, prescription opioid-related deaths totaled more than 16,000 [5]. Even in 2021, despite opiate prescribing reaching a low point, opiate deaths continue to rise [6]. Further, 1 hypothesis to explain this is that existing interventions target those who are opiate-dependent but do not prevent opioid-naïve patients from becoming addicted [7-9]. Once a patient acquires and fills a prescription, they are free to use it however they choose. The continued skyrocketing trajectory of opioid deaths as well as the persistent conundrum of opioid use demonstrates the insufficiency of exclusively relying on prescriber-directed strategies. This highlights the critical need to directly empower individual patients to responsibly manage their use, weaning, discontinuation, and disposal of opioid medications.

Continuous perioperative interventions centered on opioid management and coming from many different angles could contribute to a solution. Evidence has shown that less than 10% of unused opioids are properly disposed of after surgery, suggesting that excess opioids remaining in the home after surgery is a widespread but targetable problem [10]. Diversion of opioids, which is defined as illegal distribution or abuse of opioids for purposes not intended by the prescriber, could be intentional (for example, sharing or selling of medications) or unintentional (for example, theft or ingestion by a child).

Behavioral economics is a field of study within human decision-making where the goal is to predictably influence human behavior and encourage people to do something without forcing, coercing, or penalizing them [11,12]. Several behavioral economics interventions have been leveraged to influence human behaviors but do not guarantee an improvement in the target population’s target behavior. Nudging is a way to manipulate people’s choices to lead them to make specific decisions. Nudging has previously been used to influence smoking, alcohol consumption, diet, and physical activity to improve population health and reduce health inequalities [13]. More recently, nudging has been used to improve medication compliance among patients with various medical conditions, for example, cardiac [14], kidney transplant recipients [15], patients with diabetes [16], HIV [17], and mental illness [18]. Behavioral economics also offers an opportunity to help individuals make choices consistent with responsibly using and disposing of their opioid medications [19].

The primary goal of this project is to evaluate the effect of the 3 versions of the Opioid Management for You (OPY) tool described in the methods section on measures of opioid use relative to the standard of care (SOC) leveraging a pragmatic randomized controlled trial (RCT). We hypothesize that appropriate patient opioid use and diverted prescription opioid availability can both be improved by information delivery applied through a behavioral economics lens via sending nudges directly to the opioid users, that is, patients. We anticipate not only better management of opioid use and its safe disposal but also expect to obtain a better understanding of the unresolved questions regarding how pain medication is used in acute perioperative settings. In the future, we could further optimize the OPY tool, integrating artificial intelligence and other machine learning models for a more enhanced patient experience.

## Methods

### OPY Tool

#### Overview

A team of researchers from the Center for Learning Health System Sciences (CLHSS) at the University of Minnesota partnered with M Health Fairview (MHFV) to design, build, and test the 3 versions of the OPY tool. Based on a literature review of current popular nudging theories implemented in various delivery modes, we incorporated 3 nudging tactics into the workflow of Care Companion to improve patient self-management, medication adherence, and overall health. Tactics were based on the following behavioral economic principles: social influence, precommitment, and testimonial version. Other forms of interventions, including reminders, feedback, and loss-framing, will also be partially incorporated as a component of the design but will not be considered as major guidelines.

The tool is being built using the Epic Care Companion platform and interacts with the patient through their existing MyChart personal health record account and Epic patient portal, accessed through their phone app or the MyChart website. As patients communicate their pill usage, pain scores, or other concerns, the intervention will communicate timely and relevant information regarding opioid use and addiction while also offering encouraging feedback to help patients successfully wean and safely dispose of their opioids. OPY saves the data that a patient enters and based on their responses, the care team is alerted to important issues through an in-basket message system. The alerts or flags are selected and color-coded based on the urgency of action required after discussion with the patient education specialist team.

Each of the 3 OPY versions will deliver the same instructions and information about pain management recommendations and collect the same information about patient pain experience and side effects; however, the behavioral economics techniques used to influence weaning and disposal are different across the 3 versions. The 3 versions will correspond to different treatment arms of the RCT.

#### Version 1, Social Influence and Commitment

Social influence refers to how individuals change their ideas and behaviors to meet the needs in a social environment [20]. Studies have found that social influence has proved effective for physical activity promotion when applied to mobile apps and is also promising in helping manage chronic diseases, including diabetes.

Patients are provided with text that communicates a social norm or expresses an expectation to the patient meant to decrease the average time it takes for patients to begin weaning. Textbox 1 provides an example of social influence text shown to patients.

**Example of social influence text shown to patients.**

Opioid overdoses are at an all-time high. You can help us end this opioid epidemic! When you're ready, we'll help you wean as quickly as possible – and then get rid of your leftover medicine safely.

#### Version 2, Precommitment

Precommitment refers to a commitment we make in advance to ensure our future actions align with our current preferences and eventually reach the goal we set for ourselves [21]. Studies have shown that precommitment effectively ameliorates self-control problems by encouraging healthy diets, less smoking, and reducing temporary drinking. Being cost-effective in many cases [22], precommitment could have positive implications for chronic disease control and prevention [23].

Patients are given the same suggestions as in other versions but are asked to check a box next to the suggestions they are planning to try. Textbox 2 provides an example of precommitment options provided to patients.

**Example of precommitment options given to patients.**

**Let's try to reduce your opioid pain medicine. Set a goal to use one of the options below — and be sure to tell a friend or family member about your goal.**

- Try taking a dose every X+2 hours (instead of X hours).
- For every other dose, take just half the dose.
- Try skipping a dose.

#### Version 3, Testimonial

Emerging evidence suggests that storytelling, or narrative communication, influences listeners by actively engaging them in a story, causing them to identify themselves with the storyteller and picture themselves taking part in the action. This approach of leveraging testimonials offers a unique opportunity to promote evidence-based choices in a culturally appropriate context. Stories and storytelling have been previously used as mechanisms to improve patient medication compliance for both hypertension [24] and diabetes [25], as well as improve other health education and literacy programs.

In this approach, instead of text-based nudges or setting goals, the patient receives short videos communicating risks and benefits through narrative storytelling. The videos use voice actors to depict patients who have experienced complications related to opioid use after surgery. A video clip of a person is presented to represent the patient sharing their personal story. Textbox 3 provides an example of testimonial audio presented to patients.

To ensure that videos and their content meet the current in-place requirements of the organization, we worked closely with the patient education team and followed their requirements. We also made sure that the videos were relatable to patients from diverse demographic backgrounds (ie, race, ethnicity, gender, and age) after judicious selection of individual stock video clips from University of Minnesota stock image resources [26] and collecting voice-over audio recordings from our team members who volunteered to read and record the scripts. We will collaborate with the IT team at our implementation sites to create reports on when each patient watched the video and if they watched the full video or left without finishing it. If patients do not complete the video, the task will be flagged to ensure the patient can see and come back later to finish watching the given video.

**Example of testimonial audio presented to patients.**

After having my appendix removed, I took opioids to help with the pain. But, even on the Vicodin my doctor prescribed, I didn't feel great. When my doctor suggested that I start reducing how much I was taking, I didn't want to do that. I wasn't feeling totally better yet. When day 5 came and I hadn't reduced the number of pain pills, I had another problem: constipation. Turns out the pain of going to the bathroom was much worse than the actual pain resulting from surgery. After taking stool softeners along with gradually reducing the dose, I started feeling better in a week. I wish I had started weaning sooner.

#### OPY Functional Description

**Figure 1:**
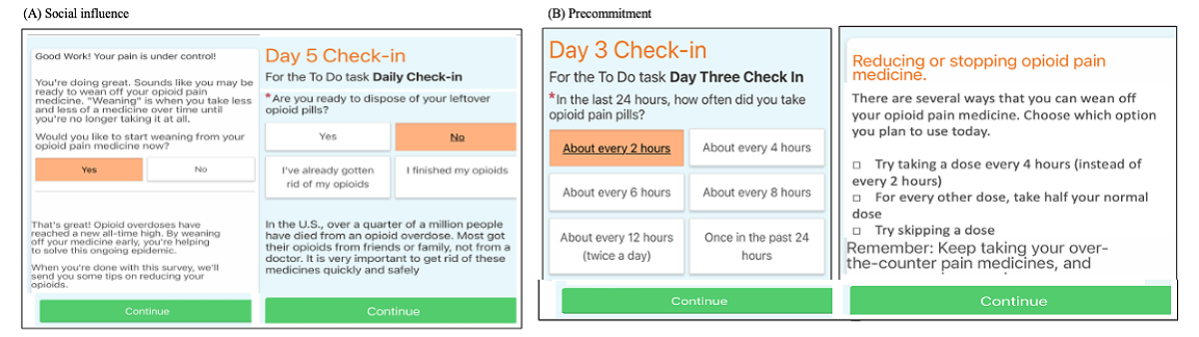
Epic Systems Corporation 2024 screenshots of OPY screen displaying questions and their responses for (A) social influence and (B) precommitment. OPY: Opioid Management for You.

OPY appears as a task within MyChart. The first OPY tasks present welcome text for first-time users that describes what OPY is and how to use it. OPY asks users if they want to use OPY or if they want to opt out. OPY tasks and questions will not appear again for people who opt out. OPY then asks patients about their comfort with filling out medical forms and their safety taking ibuprofen or acetaminophen. The medical form information is used to stratify results in our analysis, and the medication question is used to customize pain management suggestions on subsequent days. OPY users must complete these questions before receiving other content from OPY. OPY presents customized messages and questions based on the duration of time since surgery and the responses of the patient. On day 0 (the day of surgery), only the questions described will be asked. OPY patients will not know which version of OPY they are using, or that other versions exist. After day 0, a patient who has opted in will receive several questions every day offered in this order: OPY asks about side effects with prespecified suggestions for what to do for each side effect that they check, pain levels, and the number and frequency of pills they took in the last 24 hours (Figure 1).

Responses are then customized based on the number of days since surgery, patient pain level, and patient pill intake. Depending on their responses, patients receive some combination of pain management advice, including recommendations to take ibuprofen or acetaminophen if able and other standard nonmedication strategies, weaning advice on when and how to start, and disposal advice, including instructions on how and where to dispose of opioids.

At the end of the pathway, all patients will receive a patient satisfaction survey which is standard and exists as an important element of all Epic Care Companion protocols.

**Figure 2:**
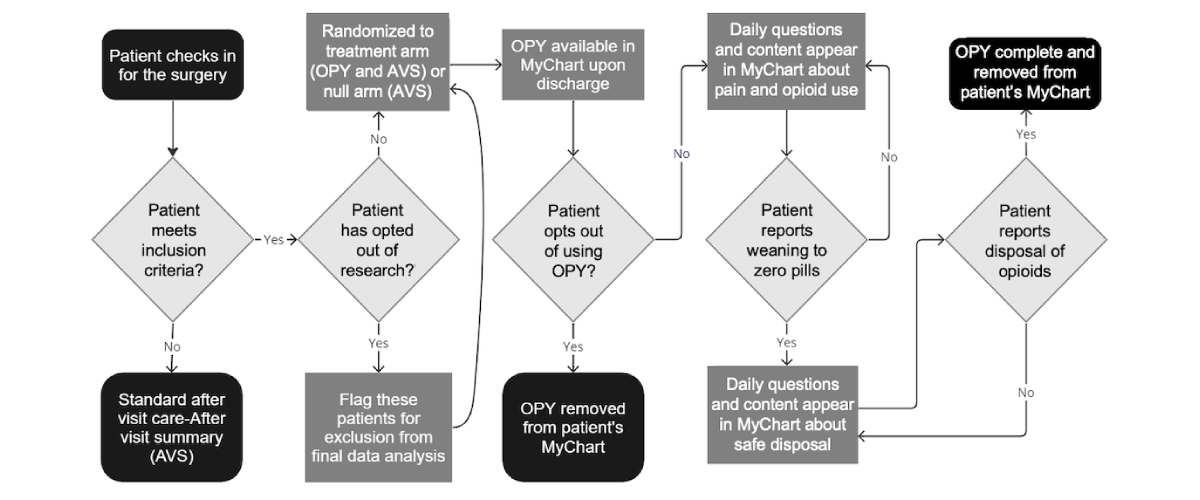
Flow of events once patients are enrolled in OPY. AVS is given to patients after medical appointments to summarize their health and guide future care. OPY: Opioid Management for You.

Once OPY patients indicate they are taking 0 opioids per day, they will be asked at most 5 times to dispose of the opioids over a span of at most 25 days. OPY will become inactive after 15 consecutive days of no response counted either from day 0 or the last response submitted. Figure 2 summarizes the flow of events that a patient encounters once they are enrolled in the OPY journey.

**Figure 3:**
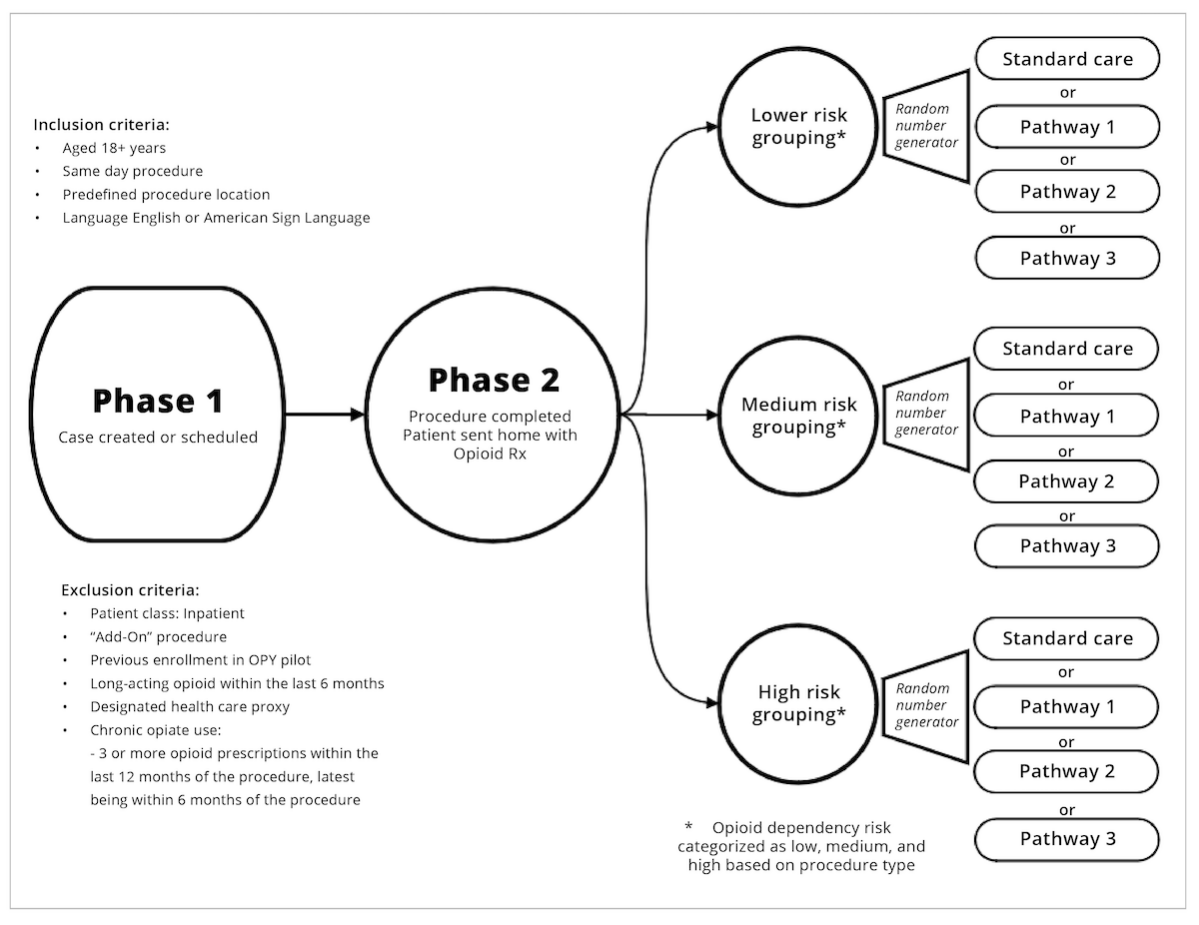
Randomization strategy. Standard care: null pathway; pathway 1: social influence; pathway 2: precommitment; and pathway 3: testimonial. First question patients receive: welcome to the Opioid Program for You! (OPY), Your care team has enrolled you in a digital opioid management program, “OPY,” … Do you want to take part in OPY? Click Yes to continue. Click No to stop getting these messages (yes or no). The number of available OPY versions will change over time, which will change the randomization ratio (eg, 1 version available=1:1, standard care versus standard care + pathway 1; 2 versions available=1:1:1, standard care versus standard care + pathway 1 versus standard care + pathway 2). We will leverage an existing random number generator that is embedded within the NAÏVE tool platform which will in turn assign a designated OPY pathway based on the ratio prescription from the statistical team. Rx: doctor’s prescription.

As per randomization strategy, patients would be assigned one of the following paths: null pathway, pathway 1, pathway 2, or pathway 3. The details are explained in Figure 3.

### Preliminary Data From the Pilot

We have demonstrated feasibility with pilot data of the OPY app (Pathway 1) by targeting our pilot to a specific cohort of patients undergoing upper-extremity procedures. About 13% of opioid-naïve patients continue to fill opioid prescriptions 90 days after hand surgery, signaling that this patient population could benefit from tools to help manage opioid use [27]. Another population that we included in the pilot is breast surgery patients, specifically opioid-naïve patients undergoing lumpectomy, mastectomy, mastopexy, and reduction mammaplasty. In total, 10% of opioid-naïve women continued to fill their prescriptions 3 months after breast reconstruction surgery [28]. Over the initial 15 weeks from August of the pilot, 100 patients were eligible to use OPY. In total, 47 (44%) of eligible patients did not interact with OPY to either opt-in or opt-out. Further, 26 (26%) patients opted out of participating in OPY and 27 (27%) opted into using OPY. There is also evidence of persistent engagement with the OPY app, with 24 (65%) of opted-in patients using the tool at least once in the first 4 days after surgery, and 16 (33%) of opted-in patients using the tool at least twice in the first 4 days after surgery as of August 10, 2023.

The preliminary data from the pilot indicates low patient engagement in continuous use. To identify the exact reason why patients were not using the OPY tool as we expected them to, we reached out to patients, their families, and even perioperative nurses. We found a few reasons, the most prominent one being introducing patients and their families to a new tool such as OPY on the day of surgery which was not considered as an effective approach. Patients are already stressed, under the influence of anesthesia, and are overwhelmed with new information. To ensure that we address those concerns, we came up with certain strategies to improve patient compliance, for example, introducing OPY during preop clinic visits, sending epic care companion messages 3 days before surgery sharing what OPY is all about, and also considering options where a nurse (who normally call on postop day 1 to ask about patient progress or pain) to remind them about OPY tool and its benefits.

### Study Design

This study will use a group-sequential RCT design to test the impact of this important health system initiative. Patients who meet OPY inclusion criteria will be stratified into low, intermediate, and high risk of opiate use based on their type of surgery. We used preliminary data to categorize surgery types according to their risk of opiate use. We ranked surgery types according to their opiate use rates at 14 days and categorized surgery types with use rates belonging to the lower 60 percentile, 60-75 percentile, and upper 25 percentile into low, intermediate, and high-risk opioid use groups (surgical risk groups in Multimedia Appendix 1). We estimate the prevalence of these subgroups to be approximately 49%, 34%, and 17%. Randomization will be stratified by baseline risk group, and, within a group, participants will be randomized equally to the SOC (the control group or nonintervention arm) or SOC plus one of the available OPY versions.

### Participant Characteristics, Sample Size, and Recruitment Strategies

#### Inclusion Criteria

We will enroll opioid-naïve patients, aged 18 years or older, including pregnant women, prisoners, and underserved populations who can read and understand English and are undergoing surgery at the MHFV Clinics and Surgery Center—Maple Grove (Maple Grove) or MHFV Clinics and Surgery Center—Minneapolis. Currently, only outpatient surgeries are performed at MHFV Clinics and Surgery Center—Minneapolis and MHFV Clinics and Surgery Center—Maple Grove. Any patient on active opioid prescription (prescribed between 30 days before surgery and until the day of surgery) who was not previously randomized or exposed to OPY would be included. All patients with cancer would be included.

#### Exclusion Criteria

We will exclude patients aged younger than 18 years; those with chronic opioid use, defined as 3 or more opioid dispensing events in the last 12 months with at least 1 of these events in the last 6 months; patients with any long-acting opioid prescription in the last 6 months; patients with a health proxy (legal guardian) designated in Epic; patients who have opted out of clinical research; and patients with an active palliative care referral.

Children are excluded due to complexities in postoperative care and communication.

#### Sample Size

The study will enroll up to 3500 participants (approximately 1715, 1190, and 595 enrollments to the low, intermediate, and high-risk groups, respectively) in each OPY version.

#### Recruitment

We will recruit patients who are undergoing surgery with participating providers at the 2 predetermined locations, that is, MHFV Clinics and Surgery Center in Minneapolis and the Maple Grove locations. Patients who opt out of clinical research or for whom opt-out status is missing will be excluded. The patient is given a choice to consent or opt out of the OPY journey during their first postoperative interaction with the OPY questionnaire distributed through Epic Care Companion. The final decision to participate or not in the OPY journey would be collected from the patient on the day of the surgery (end of the day) through the OPY questionnaire distributed through Epic Care Companion leveraging MyChart.

### Study Duration and Data Management

We anticipate that the pragmatic trial will be implemented over 24 months. Our trial will continue enrollments until we reach our 3500 patients enrollment goal. We rely entirely on collaboration with MHFV for data collection within the CLHSS and Fairview data specialist teams. This is because these efforts require the creation of new data fields that leverage a learning health system platform. Unless specified, we will use naturally collected NAÏVE data (including OPY usage) to assess process fidelity, patient safety, and impact on outcomes.

This project will use the existing CLHSS or Center for Quality Outcomes, Discovery, and Evaluation database covered by the institutional review board (IRB) under 597 Protocol (STUDY00014481). The research database lives within Fairview IT. At Fairview, IT access is facilitated by Fairview Research. Please see the data governance processes described in IRB 597 Protocol (STUDY00014481) for further details. This study will rely on the surgical data mart for data acquisition outside of (1) the data primarily generated through the patients’ clinical participation in the OPY tool and (2) follow-up surveys.

Data will not be shared publicly. Access is regulated by the CLHSS and Fairview Data stewards. Additional sharing beyond the focus of this study would require additional IRB approval.

### Data Collection

Patients meeting OPY inclusion criteria will receive a MyChart message notifying them of access to a specific OPY app. Each patient, regardless of assignment to an OPY version, will receive SOC information via the after-visit summary regarding the appropriate use and disposal of opiates and how to contact their care team if pain persists or worsens. Implementation of each app is primarily driven by the health system. The research team will assist with the allocation approach and subsequent analysis. OPY will collect and send data via the Epic care companion platform leveraging MyChart. Data generated through patient interactions with the OPY tool will live in the Epic environment while the patient is interacting with the tool. This includes data such as daily pain scores, daily opiate usage, daily deferrals of weaning, use of nonopiate medications, “red flag” triggers, and disposal of excess medication. Data generated through interaction with the tool will subsequently be transferred to an Academic Health Center Information Exchange–compliant server as described above. Data on various stakeholder (patients and clinicians) perspectives about their experience with the OPY tool or program would be collected periodically using surveys and or interviews.

### Primary and Secondary Study Outcomes

Our primary outcome is the persistent use of opiates as measured by continued use at 14 days. Secondary end points we will seek to evaluate include the number of days between initiation of opioid therapy and opioid-free pain control; the interval time between opioid doses; daily pain scores; number and cause of patient-initiated outreach events (from the care companion app); the number of MyChart messages within the first 30 days postoperative; the number of phone notes in the first 30 days postoperative; 90-day hospitalization rates and hospital length of stay; repeat surgery rates; outpatient encounter rates; referral and completion of referral of pain management rates; 90- and 120-day opiate use rates; OPY usage at 1, 3, 7, 14, and 30 days, 90 days all-cause mortality; metrics around patients’ reported disposal of remaining pills, for example, time to disposal and preferred disposal route.

Outcome data will be extracted from natural electronic health record data, common to pragmatic trials. Specific measures including tool usage, opioid prescribing rates, and the existence of comorbidities, are all validated as a part of quality reporting (opioid reduction optimal care map). Each measure is validated at the time of variable construction and immediately before statistical analysis.

### Data Analysis

The analysis of the primary and secondary end points will be completed following the intention-to-treat principle. Participant data will be analyzed according to the randomly assigned treatment group. We will ensure that only records of patients who have agreed to have their information used for research are included in the analysis. We will include all patients who opted into the OPY experiment whether they ever used the OPY tool or not.

The baseline opioid use rate of patients varied substantially between the low, intermediate, and high-risk groups. Moreover, preliminary data suggested that our new tool’s effectiveness may vary strongly across subgroups. Therefore, we opted out of pooled analyses including all 3 subgroups. Instead, we will conduct 3 prespecified subgroup analyses and each will use a 5% type I error level.

### Efficacy Analysis

The study will enroll up to 3500 participants (approximately 1715, 1190, and 595 enrollments to the low, intermediate, and high-risk groups) in each OPY version. Interim and final analyses efficacy analyses will be conducted every 3 months, separately for each surgery risk group, using an O’Brien-Fleming type error-spending function approach to account for the multiple interim and final analyses. Risk-group-specific group-sequential hypothesis tests will use a 1-sided 5% type I error level, to control the overall type I error for each of the 3 interventions at a 15% type I error level.

### Futility Interim Analyses

Futility interim analysis will be conducted every 3 months separately in each risk group. The study will stop testing an OPY version in a subgroup if, at the interim analysis, the predicted probability of a positive study outcome (predicted power) in this subgroup drops below 15%.

### Power Analysis

Based on our initial estimates of the 14-day opioid use rates (0.03, 0.07, and 0.16) for the SOC in each of the 3 subgroups and an odds-ratio treatment effect of 2.0 (opioid use rates 0.015, 0.037, and 0.087), the group-sequential design has 64%, 82%, and 85% power to detect positive treatment effects in the low, intermediate, and high-risk subgroups.

### Study Risks

In this minimal-risk study, there is a very low risk to participants. This risk extends to providers involved in the study or family members of participants. Breach of confidentiality is unlikely but remains a possibility. Data will be maintained in a secure, HIPAA (Health Insurance Portability and Accountability Act)-compliant environment.

### Ethical Considerations

This study protocol is submitted to the University of Minnesota IRB and given the initial determination of “Not human subjects research.” All patients will be e-consented to the study before data collection. This will be verified by study staff before data collection and analysis. No data from patients outside the study group will be transmitted into the study database. The study obtained IRB approval on 31 August, 2023 (STUDY00019820). The trial was registered in ClinicalTrials.gov (NCT06124079).

## Results

This study was originally started in August 2022 and is being supported by the Rapid Prospective Evaluation and Digital Technology Innovation programs at CLHSS. The implementation of this intervention has been fully resourced by MHFV. The structure of engagement, survey development, data collection, statistical analysis, and dissemination will be coordinated by the Rapid Prospective Evaluation Program and funded entirely. The pilot was launched in February 2023 and is still running. Preliminary data from the pilot collected in August 2023 is reported in the section Methods: Preliminary Data From the Pilot. The RCT is planned to start by the end of August 2023.

## Discussion

### Principal Results

In this paper, we present the protocol for the OPY study, a group-sequential pragmatic RCT design of patient-facing digital tools deployed using their existing chart personal health record platform. The purpose of this innovative tool is to effectively manage opioid use, weaning, and disposal among postoperative patients by directly interacting at patient levels leveraging behavioral economics principles. To our knowledge, this is one of the first-ever pragmatic clinical trials to study the impact of introducing behavioral economics techniques into a patient-facing, innovative digital technology on opioid management, leveraging the learning health system. The trial is set up as a hybrid implementation-effectiveness trial with strategies informed by behavioral economics theory.

The efficacy of artificial intelligence chatbots in promoting healthy lifestyles, smoking cessation, treatment or medication adherence, and reduction in substance misuse has been tested earlier; however, there were mixed results regarding feasibility, acceptability, and usability [17]. OPY is a novel solution to aid with weaning and disposal of opioids in opioid-naïve people and acts uniquely in a preventative manner. By using an OPY tool, equivalent to an archetype of a chatbot, we could integrate a natural language interface, delivering the pertinent information to the postoperative patients around opioid use at the relevant times and reporting graded alerts or flags to clinical staff to initiate a patient or provider communication if necessary. Earlier evidence has shown that patient characteristics (such as age and gender) play a pivotal role in determining the probability of filling an opioid prescription [3,18,19]. With the implementation of this patient-facing OPY tool, we could help postoperative, naïve patients who are at risk of addiction to more effectively manage these medicines.

By combining the OPY tool, a preliminary version of a chatbot, with behavioral economics, we can use social influence (social norms), precommitment, and testimonial-based “nudges” to encourage patients toward more responsible opioid usage. Future nudges may include social norms, loss aversion, salience effect, and IKEA effect to encourage patients [20]. In the future, we could include infographics showing harm from inappropriate opioid storage and disposal and create charts for patients on opioid use to reinforce patient decisions.

There is some evidence that the effect of behavioral economics nudges on human behavior is not guaranteed to have the desired effects. Introducing behavioral nudges in some instances, has induced the opposite behavior than desired [29-31]. For example, precommitment can unintentionally telegraph to the patient that the behavior has low urgency, decreasing their motivation to accomplish the task. Another well-established mechanism for influencing target behaviors is communicating social norms that support the intended behavior. Social norm nudges are generally accepted to operate by creating some amount of discomfort within the patient, by demonstrating that they are not doing what is typically done by others, to encourage the intended behavior [32]. Our study will help clarify if the behavioral economics techniques we have incorporated can inspire early weaning and disposal or not.

### Limitations

A limitation of this study is that the app is currently only available in English, and we will only include patients who can read and write basic English. We are also excluding patients lacking the capacity to consent, as well as children, due to their inability to functionally and meaningfully interact with the app. We will only limit the enrollment to patients undergoing same-day surgery across 2 locations and not system-wide at this time. This limits the operational lift to execute this project and the number of staff needing new training on the OPY technology and care pathway. We do plan to expand to outpatient surgery at the hospital and then to inpatients. Further, in the testimonial version of OPY, the images or voices over the image are generic and the current technology does not have the capability of matching with the patient’s actual demographics.

### Conclusions

In summary, by implementing OPY in opioid-naïve patients, we will be able to help prescribers remotely manage new opioid prescriptions. Given the significant limitation in physical proximity with the care team, we must empower patients with a practical tool to assist in weaning off and disposing of opioid medications that are available to them 24/7. Implementation of 3 versions of OPY, all designed to promote weaning from opioids and legal disposal in opioid-naïve patients, will produce generalizable evidence about the impact of behavioral economics on patient prescription behavior. If successful, OPY will advance the understanding and effectiveness of electronic health record–based strategies with patient interaction to improve the delivery of evidence-based care to patients at high risk for opioid addiction.

## Appendix

Multimedia Appendix 1 Ranking of surgery types according to their opiate use rates at 14 days. Surgeries are listed from lowest to highest rates of opiate use. Surgery types are additionally categorized by use rates into lower 60 percentile (low-risk opioid use group), 60-75 percentile (medium-risk opioid use group), and upper 24 percentile (high-risk opioid use group).

## Abbreviations

CLHSS: Center for Learning Health System Sciences
HIPAA: Health Insurance Portability and Accountability Act
IRB: institutional review board
MHFV: M Health Fairview
OPY: Opioid Management for You
RCT: randomized controlled trial
SOC: standard of care
